# Relationship between Medication-Related Osteonecrosis of the Jaw and CDK4/6 Inhibitors in Breast Cancer

**DOI:** 10.3390/curroncol31010016

**Published:** 2024-01-01

**Authors:** Loreto Domínguez Senín, David Morales Pancorbo, María Yeray Rodríguez Garcés, María Dolores Santos-Rubio, Juan Bayo Calero

**Affiliations:** 1 Hospital Pharmacy, Juan Ramón Jiménez University Hospital, 21005 Huelva, Spain; mariad.santos.sspa@juntadeandalucia.es; 2 Department of Medical Oncology, Juan Ramón Jiménez University Hospital, 21005 Huelva, Spain; david.morales.pancorbo.sspa@juntadeandalucia.es (D.M.P.);; 3 Department of Medical Oncology, Riotinto Hospital, 21660 Huelva, Spain

**Keywords:** MRONJ, CDK4/6 inhibitors, metastatic breast cancer, oral epidemiology

## Abstract

Objective: We aimed to evaluate the use of CDK4/6 inhibitors as a risk factor for medication-related osteonecrosis of the jaw (MRONJ) in a cohort of patients with metastatic breast cancer treated with denosumab. Methods: This was a multicentre, retrospective, observational study. All patients with breast cancer treated with denosumab (January 2011–December 2022) were included. The relationship between CDK4/6 inhibitors and MRONJ was analysed. Results: A total of 243 patients were included, ninety-five (44.2%) of whom used a CDK4/6 inhibitor. There were 21 patients with MRONJ. In patients treated with denosumab without CDK4/6 inhibitors, the incidence of MRONJ and mean time to the occurrence of MRONJ were 6.6% (8/120) and 16.8 months (SD 7.8), respectively; in patients treated with denosumab and CDK4/6 inhibitor, these values were 13.7% (13/95) and 15.4 months (SD 8.7), respectively. The difference in the incidence was not significant (*p* = 0.085). Among the 19 patients who used abemaciclib, the probability of MRONJ occurrence was significantly higher compared to patients not using CDK4/6 inhibitors (*p* = 0.0178). Conclusions: These results suggest that the incidence of MRONJ in patients with metastatic breast cancer treated with denosumab is higher, and the onset of MRONJ occurs earlier in the presence of CDK4/6 inhibitors. The differences were statistically significant in the patients who used abemaciclib. Given that the use of this combination is very common in routine clinical practice, it would be advisable to carry out larger prospective studies to clarify the risk of this association.

## 1. Introduction

In 2002, medication-related osteonecrosis of the jaw (MRONJ) was first described as a relatively uncommon but potentially serious side effect. It is related to osteoclast inhibitors therapy such as intravenous, high-potency bisphosphonates and denosumab [1].

Denosumab is a human monoclonal antibody, namely immunoglobulin G2 (IgG2), that targets and binds with high affinity and specificity to the receptor activator for nuclear factor κ B ligand (RANKL), preventing the interaction of RANKL/RANK from occurring and causing a reduction in the number and function of osteoclasts. The inhibition of the RANKL–RANK interaction impedes osteoclast formation, function, and survival, thereby decreasing bone resorption [2]. Bisphosphonates are analogues of pyrophosphate in which a carbon replaces the central oxygen. Bisphosphonates inhibit osteoclast activity, thereby decreasing bone resorption and increasing bone mineralization [3,4]. Bisphosphonates act as potent inhibitors of bone resorption because they have a direct apoptotic effect on osteoclasts and affect osteoclast differentiation and maturation. Bisphosphonates are used in the prevention of events related to the skeleton (pathological fracture, radiotherapy, compression of the spinal cord, or bone surgery) in adults with advanced neoplasms with bone involvement.

Denosumab 120 mg subcutaneously injected every four weeks is the approved dosage for the prevention of skeletal-related events in patients with bone metastases from solid tumours. At this dosage, the risk of MRONJ is consistently slightly higher than that observed with intravenous bisphosphonates [5,6,7].

After recognition of jaw necrosis as a complication of other drugs, a special committee of the American Association of Oral and Maxillofacial Surgeons (AAOMS) recommended “MRONJ” as a preferred treatment [8]. The Multinational Association of Supportive Care in Cancer (MASCC)/International Society of Oral Oncology (ISOO)/American Society of Clinical Oncology (ASCO) includes this terminology in their joint guidelines [9].

The diagnosis of MRONJ is characterized by current or previous treatment with an osteoclast inhibitor or an antiangiogenic agent, exposed or necrotic bone in the maxillofacial region that has persisted for more than eight weeks, and no history of radiation therapy or obvious metastatic disease in the jaw bones [8]. The underlying pathophysiology has not yet been fully clarified. The main hypotheses proposed to explain MRONJ include impaired bone repair and osteoclast activity suppression; impaired angiogenesis or vascular repair; and local factors such as poor dental hygiene, advanced periodontal disease, poorly fitting dentures, and/or some type of dental manipulation (e.g., tooth extraction) [10,11,12,13,14,15,16]. However, no hypotheses seem to explain all cases [17].

Before the development of clinically detectable osteonecrosis, there could be signs and symptoms. These possible signs and symptoms are prolonged jaw pain, tooth mobility, bone enlargement, gingival inflammation, erythema, and ulceration [18,19,20]. According to some reports, once MRONJ develops, better results may be obtained when osteoclast inhibitor therapy is discontinued for one to six months [21,22]; however, discontinuation of these agents could also lead to the recurrence of bone pain, the progression of metastases, and/or increased skeletal pain. This issue was addressed in the 2019 MASCC/ISOO/ASCO joint guidelines, which state that the decision is left to the discretion of the physician after a discussion with the patient and/or caregiver [9].

In 2020, Marcianò et al. [23] reported a possible association between MRONJ and cyclin-dependent kinase (CDK) 4/6 inhibitors in breast cancer patients receiving osteoclast inhibitor therapy.

In combination with an aromatase inhibitor or fulvestrant, CDK4/6 inhibitors (palbociclib, ribociclib, and abemaciclib) have been approved by the Food and Drug Administration (FDA) for the treatment of hormone receptor (HR)-positive, growth-factor-receptor-2-positive, locally advanced, or metastatic human epidermal (HER2)-negative breast cancer [24,25,26].

Up to 25% of patients with localized disease at diagnosis may develop metastases, with bone being the most common site. A total of 70 to 90% of patients with breast cancer have some form of skeletal metastasis [27,28]. In patients with breast cancer with bone metastases, CDK4/6 inhibitors and osteoclast inhibitors are concomitantly used for their respective indications. Furthermore, considering that bone metastasis is the most frequent form of presentation in luminal metastatic breast cancer and considering the efficacy of CDK4/6 inhibitors, the use of both drugs over a long period is foreseeable.

The work by Marcianò et al. consists of the description of only six cases of MRONJ in patients with breast cancer treated with osteoclast inhibitor therapy and CDK4/6 inhibitors among a total of sixteen cases of MRONJ. In 2023, a description of eight cases of MRONJ in patients with palbociclib was published without finding a specific pattern that could suggest a triggering role of palbociclib in the development of MRONJ [29].

Therefore, the aim of this study was to evaluate the use of CDK4/6 inhibitors as a risk factor for MRONJ in a cohort of patients with metastatic breast cancer treated with denosumab. The demographic and clinical variables of the patient cohort were analysed, and the duration of treatment with denosumab and CDK4/6 inhibitors was calculated. The cases of MRONJ in the cohort were described. In this group, in addition to the above variables, the time from the start of denosumab to the onset of the event and the risk factors already described for developing MRONJ were reported.

## 2. Materials and Methods

### 2.1. Patients

This was a multicentre, retrospective, observational study. Data were collected for all patients who had been treated with denosumab for metastatic breast cancer at two hospitals, namely Juan Ramón Jiménez Hospital and Riotinto General Hospital, in the province of Huelva, Spain. Data were collected from January 2011, the year in which the drug was marketed, to December 2022, i.e., the end date of the study. All patients with a biopsy-confirmed diagnosis of advanced or metastatic breast cancer with bone involvement who were attending medical oncology follow-ups and who had received at least one dose of denosumab 120 mg subcutaneously for the prevention of bone events were included.

Patients for whom access to their electronic medical records was not granted, patients who did not attend medical oncology follow-ups or did not obtain their medication from the hospital pharmacy service, and patients who received only a single cycle of CDK4/6 inhibitors were excluded.

A research protocol with concrete methodological guidelines was developed. Data sources were identified, and their appropriateness for the aims of the study was verified. The research protocol was presented to the province’s biomedical research ethics committee.

Clinical information was obtained from the electronic medical records of the patients, and medication-dispensing data were obtained from the Athos^®^ Prisma program used in the hospital pharmacies for outpatient consultations.

### 2.2. Treatment and Assessment

The dosage of denosumab used to prevent bone events was 120 mg administered as a single subcutaneous injection once every 4 weeks. Dose adjustments were not necessary for patients with renal insufficiency or elderly individuals.

Abemaciclib was used at a dosage of 150 mg twice daily combined with hormonal therapy as long as the patient obtained clinical benefits with no toxicity. The ribociclib dosage was 600 mg (three 200 mg film-coated tablets) once daily for 21 consecutive days, followed by 7 days without treatment, in cycles of 28 days. Palbociclib was used at a dosage of 125 mg per day in combination with an aromatase inhibitor or fulvestrant. In cases of toxicity, the doses were reduced based on indications in the technical data sheets.

To be considered a case of MRONJ, this diagnosis had to be described in the maxillofacial surgery or medical oncology medical history and confirmed by an oncologist or maxillofacial surgeon via clinical or radiological findings. The American Association of Oral and Maxillofacial Surgeons’ Position Paper on Medication-Related Osteonecrosis of the Jaw—2022 Update was used to classify MRONJ [30]. This document describes a staging system for MRONJ that describes stage 0 (non-specific symptoms or clinical and radiographic findings but without clinical evidence of necrotic bone), stage 1 (asymptomatic patients with exposed and necrotic bone or a fistula that penetrates the bone without evidence of infection/inflammation; these patients may also present radiographic findings for stage 0 that are localized in the region of alveolar bone), stage 2 (symptomatic patients with exposed and necrotic bone or a fistula that penetrates the bone with evidence of infection/inflammation; these patients may also present with the aforementioned radiographic findings for stage 0 localized in the region of alveolar bone), and stage 3 (symptomatic patients with exposed and necrotic bone or fistulas that penetrate the bone with evidence of infection and one or more of the following: necrotic exposed bone extending beyond the alveolar region, pathological fracture, extraoral fistula, oral–nasal communication, or osteolysis that extends to the lower edge of the jaw or the floor of the sinus).

Demographic and clinical variables (e.g., age, sex, comorbidities (arterial hypertension (HT), diabetes, and lipid disorders), and extraosseous metastases) of the patients were extracted from their clinical histories. The duration of treatment with denosumab and CDK4/6 inhibitors was calculated. For patients with MRONJ, the stage, time from the start of denosumab treatment to the onset of the event, and risk factors for developing MRONJ (poor dental hygiene, advanced periodontal disease, poorly fitting dentures, some type of dental manipulation, and use of other drugs related to the appearance of MRONJ) were recorded.

### 2.3. Statistical Analysis

The deadline for including data in the analysis was December 2022. The incidence of MRONJ was determined for the group of patients treated with denosumab and CDK4/6 inhibitors and for the group of patients treated with denosumab without CDK4/6 inhibitors. Chi-square analysis was used to compare the incidences of MRONJ in both groups and determine whether there was a significant difference in the occurrence of MRONJ in both groups.

A descriptive analysis of demographic data, patient characteristics, cancer treatments, and the duration of cancer treatments was performed. Continuous data are presented herein as the mean (standard deviation) or median (range), and categorical data are presented as the frequency and proportion. Cases of MRONJ are described.

### 2.4. Ethics Statement

The authors are accountable for all aspects of the present work. Any questions related to the accuracy or integrity of any part of this work have been appropriately investigated and resolved. Informed consent was not needed for participation because this investigation was a retrospective study. All authors had full access to all the data in the study. The procedures were conducted in accordance with the precepts of good clinical practice and the Declaration of Helsinki.

## 3. Results

A total of 243 patients undergoing denosumab treatment for metastatic breast cancer at Juan Ramón Jiménez Hospital and Riotinto General Hospital in the province of Huelva were included from study inception until December 2022. Of the total, 28 patients were excluded: 19 because of no access to their electronic medical records and 9 because of a single cycle of CDK4/6 inhibitors.

Of the 215 patients included, 95 (44.2%) used CDK4/6 inhibitors: 19 (20%) used abemaciclib, 41 (43.1%) used palbociclib, 29 (30.5%) used ribociclib, and 6 (6.3%) used more than one CDK4/6 inhibitor. A total of 120 patients (55.8%) did not use any CDK4/6 inhibitor. There were 21 patients with MRONJ. Of these patients, 13 used CDK4/6 inhibitors (5 used abemaciclib, 5 used palbociclib, and 3 used ribociclib). The demographic and clinical characteristics of the patient group are shown in Table 1. All patients were women, and the median age was 59 years (range: 29–88). A total of 105 patients (48.8%) presented with extraosseous metastases, and the most frequent comorbidity was hypertension (38.1%; 82 patients).

The mean duration of denosumab treatment was 14.3 months (standard deviation, 9.6 months). The mean duration of treatment with any CDK4/6 inhibitor was 15.3 months (standard deviation, 10.9 months); the mean duration of treatment was 14.3 months for abemaciclib (standard deviation, 9.9 months), 17.11 months for palbociclib (standard deviation, 12.2 months), and 15 months for ribociclib (standard deviation, 10.3 months). By the end of the study, 57.7% (n = 124) of the patients had died.

The incidence of MRONJ was 6.6% (8/120) in patients treated with denosumab without CDK4/6 inhibitors and 13.7% (13/95) in patients treated with denosumab with a CDK4/6 inhibitor. Although the group treated with CDK4/6 inhibitors had a higher incidence of MRONJ cases, the difference in incidence between the groups was not significant (*p* = 0.085).

Among the nineteen patients who used abemaciclib, five experienced MRONJ. The probability of MRONJ occurrence was significantly higher compared to patients not using CDK4/6 inhibitors (*p* = 0.0178 by Fisher’s exact test) and also significantly higher compared to all other patients (*p* = 0.0237 by Fisher’s exact test).

For patients with MRONJ (21 patients), 9 had stage 0 MRONJ, 7 had stage 1 MRONJ, 5 had stage 2 MRONJ, and 0 had stage 3 MRONJ. The median age was 60 years (range 46–83), 100% (21/21) were women, and 33.3% (7/21) presented extraosseous metastases. The most frequent comorbidity in this group was hypertension (n = 6), followed by lipid disorders (n = 4) and diabetes (n = 2). In the Table 2 are listed all patients with MRONJ.

The mean duration of treatment with denosumab in these 21 patients was 16.8 months (standard deviation, 9.3), and the mean duration of treatment with CDK4/6 inhibitors was 18.9 months (standard deviation, 6.2). The mean time from the start of denosumab to the onset of the event was 15.4 months (standard deviation, 8.7) for the 13 patients who used denosumab and CDK4/6 inhibitors and 16.8 months (standard deviation, 7.8) for the 8 patients who used only denosumab.

Patients in this group had risk factors for developing MRONJ: three patients had poor dental hygiene, two patients had advanced periodontal disease, and five patients had poorly fitting dentures and/or some type of dental manipulation. Regarding the use of other drugs related to the occurrence of MRONJ, two patients with MRONJ had previously used bevacizumab (3 cycles and 12 cycles), and one patient with MRONJ had used everolimus for 11 months.

## 4. Discussion

The association between CDK4/6 inhibitors and MRONJ was suggested for the first time by Marcianò et al. Although no mechanism of action has been described, Marcianò et al. associated stomatitis/mucositis produced by CDK4/6 inhibitors with an eventual risk of developing MRONJ. Stomatitis is described in pivotal studies (palbociclib—PALOMA; ribociclib—MONALEESA; and abemaciclib—MONARCH) as an adverse effect associated with the use of the three CDK4/6 inhibitors. Studies that evaluated palbociclib reported low occurrences of stomatitis, with 30% vs. 14% and 28% vs. 13% in the PALOMA-2 trial and in the phase III PALOMA-3 trial, respectively [31]. In the MONARCH-2 trial, the incidence of all-grade oral mucositis (OM) during abemaciclib treatment was low (15% vs. 10%), with 1% ≥ G3 OM [32,33]. In stomatitis, there is a breakdown of the lining of the mucosa in the mouth and therefore exposure of the underlying bone to bacteria. This scenario is why Marcianò et al. linked stomatitis/mucositis produced by CDK4/6 inhibitors with an eventual risk of developing MRONJ.

Later, Fusco V et al. [34] published a comment indicating that the study by Marcianò et al. lacked consistency because it described six cases of MRONJ in patients with breast cancer treated with osteoclast inhibitors and CDK4/6 inhibitors among a total of sixteen cases of MRONJ in patients at a reference centre for oral care.

In this study, all patients with metastatic breast cancer who received denosumab were included to compare the incidence of MRONJ between patients treated with CDK4/6 inhibitors and osteoclast inhibitors and those who only received osteoclast inhibitors. The incidence of MRONJ was found to be higher in the group of patients treated with CDK4/6 inhibitors (13.7%) than in the group of patients who were not treated with CDK4/6 inhibitors (6.6%). Among the patients who used abemaciclib, the probability of MRONJ occurrence was significantly higher compared to patients not using CDK4/6 inhibitors and also significantly higher compared to all other patients. This suggests that within CDK4/6 inhibitors, abemaciclib might be the most influential in terms of MRONJ occurrence.

In 2023, Chabnam Y et al. [29] analysed a retrospective case series of patients treated with palbociclib between 2016 and 2020 at the Rennes Dental Care Center. They found eight cases with a diagnosis of osteonecrosis of the jaw. All patients had at least one risk factor (dental extraction, dentures, and denosumab exposure at the time of diagnosis). They did not identify a specific pattern that could suggest a triggering role of palbociclib in the development of osteonecrosis of the jaw.

The incidence of MRONJ in patients treated with denosumab without CDK4/6 inhibitors was 6.6%. Some data support the view that the risk of MRONJ with denosumab stabilizes between 2 and 3 years [35,36]. In an analysis of three registered phase III trials, the incidence of developing MRONJ was 1.1% during the first year and 4.1% thereafter [5]. Based on the results of the extension phases of two phase III studies, the risk of MRONJ with denosumab was 1.1% during the first year, 3.7% during the second year, and 4.6% per year thereafter [37]. According to Stopeck AT et al., the patient incidence of ONJ during the open-label extension phase (not adjusted for years of patient follow-up) was 6.9% in the denosumab/denosumab group. A history of tooth extraction, poor oral hygiene, and/or the use of a dental appliance was reported for 93% of patients who developed MRONJ [37]. The high incidence in our group of patients could be attributed to poorer dental hygiene and a history of extractions. According to the 2020 European Health Survey, the perceived oral health status in Spain compared with that in other European countries is poor or very poor [38].

In the patient group treated with denosumab and a CDK4/6 inhibitor, the incidence of MRONJ was 13.7%. No studies in the literature have compared the rate of MRONJ between patients treated with CDK4/6 inhibitors and osteoclast inhibitors and that in patients receiving only osteoclast inhibitors. In this study, the incidence was higher in the group of patients treated with CDK4/6 inhibitors; although the difference was not significant, the incidence in this group was very high.

The demographic and clinical variables were similar between the two groups analysed.

The mean duration of denosumab treatment was 14.3 months, which is within the recommended range. There are no data on the optimal duration of osteoclast inhibitor therapy. The American Society of Clinical Oncology (ASCO) and the European Society of Medical Oncology (ESMO) recommend continuing treatment indefinitely in the absence of excessive toxicity and provided that the treatment is agreed upon by the patient and meets the objective of medical treatment [39,40]. Some guidelines have proposed use for up to two years. The 2020 European Society for Medical Oncology (ESMO) guidelines suggest that bisphosphonate therapy can be discontinued after 2 years for patients with oligometastatic disease who are in remission. The ASCO guidelines on bone modifying agents in multiple myeloma similarly suggest therapy for two years and reassessment over time [41]. The long-term efficacy and toxicity of long-term osteoclast inhibition (i.e., more than two years) requires further investigation in prospective trials.

The mean duration of treatment with CDK4/6 inhibitors was 15.3 months. Palbociclib was approved by the FDA based on a phase III study [24] in which the combination of palbociclib with letrozole resulted in a progression-free interval (PFI) of 24.8 months (95% CI: 22.1, NE). Ribociclib in combination with letrozol was approved by the FDA based on a phase III study [25] in which the PFI was 25.3 months (95% CI: 23.0–30.3). In the MONARCH 3 trial [26], the combination of abemaciclib with an aromatase inhibitor (letrozol or anastrozol) resulted in a PFI of 25.3 months (95% CI: 23.0–30.3). The objective of this study was not to assess the efficacy of CDK4/6 inhibitors (PFI or overall survival); therefore, the reasons for the discontinuation of treatment with CDK4/6 inhibitors (disease progression, death, toxicity, or voluntary suspension) were not determined. However, the average duration of treatment with CDK4/6 inhibitors was acceptable in this study because there was a group of women treated with second or successive lines, which would justify the lower PFI obtained.

In the group of patients with MRONJ, the mean durations of treatment with denosumab and CDK4/6 inhibitors (16.8 and 18.9 months, respectively) were acceptable.

Using the 2022 update on the classification of MRONJ, 9 out of 21 (42.9%) patients had stage zero MRONJ, without clinical evidence of necrotic bone but with nonspecific symptoms or clinical and radiographic findings. This finding increases the incidence of MRONJ. MRONJ onset occurred earlier in the group treated with CDK4/6 inhibitors (15.4 months) than in the group treated without CDK4/6 inhibitors (16.8 months). According to the technical data sheet, the mean time to ONJ is 18.7 months (range, 1–44 months).

Regarding the related risk factors, the use of antiangiogenic agents together with an osteoclast inhibitor for the prevention of skeletal-related events seems to increase the incidence of MRONJ [42,43,44,45,46,47,48,49,50]. There are an increasing number of reports of MRONJ in patients treated with a variety of antineoplastic drugs without the concomitant use of an osteoclast inhibitor, including inhibitors of mechanistic (previously called mammalian) target of rapamycin (mTOR), BRAF inhibitors, and immunotherapy; most cases are in patients receiving angiogenesis inhibitors. In a review of 42 cases of MRONJ related to nonosteoclastic inhibitor therapy reported in the literature, 31 were in patients who received angiogenesis inhibitors alone or in combination [51]. The use of these drugs was not significant in our group; only three patients were treated with such drugs, one of whom received only three cycles of bevacizumab.

A limitation of this study was its retrospective design. Although the number of patients was limited, this is the largest study to explore the relationship between MRONJ and treatment with CDK4/6 inhibitors. There were also some difficulties in terms of accessing medical records, some of which were in physical and not electronic format because the digitization of medical records occurred after the commercialization of denosumab.

## 5. Conclusions

The results of this study suggest that the incidence of MRONJ in patients with metastatic breast cancer treated with denosumab is higher and occurs earlier in the presence of CDK4/6 inhibitors. The differences were statistically significant in the patients who used abemaciclib. Given that the use of this combination is very common in routine clinical practice, it would be advisable to carry out larger prospective studies to clarify the risk of this association.

## Figures and Tables

**Table 1:** Demographic and clinical characteristics of the two groups of patients.

	Denosumab N = 120			Denosumab + CDK4/6 Inhibitors N = 95		
**Mean age (years)**	61.7			58.2		
**Sex (n (%))**	**Female** 120 (100)		**Male** 0 (0)	**Female** 95 (100)		**Male** 0 (0)
**Visceral metastasis (n (%))**	**Yes** 64 (53.3)		**No** 56 (46.7)	**Yes** 41 (43.2)		**No** 54 (56.8)
**Comorbidities (n (%))**	**AHT** 49 (40.8)	**DL** 39 (32.5)	**MD** 15 (12.5)	**AHT** 33 (34.7)	**DL** 18 (18.9)	**MD** 11 (11.6)

AHT, arterial hypertension; DL, dyslipidaemia; MD, diabetes mellitus.

**Table 2:** Patients with MRONJ.

	Sex	Age	Duration of Denosumab Treatment (Months)	CDK4/6 Inhibitor	Duration of CDK4/6 Inhibitor Treatment (Months)	Comorbidities	Visceral Metastasis	Time from the Start of Denosumab to the Onset of the Event	Dental Osteonecrosis Risk Factor
**Patient 1**	Female	65	16	Palbociclib	19	No	No	16	Poor dental hygiene
**Patient 2**	Female	55	21	No		No	Yes	6	Advanced periodontal disease
**Patient 3**	Female	51	11	Ribociclib	12	No	No	11	
**Patient 4**	Female	78	7	Abemaciclib	22	HTA	No	7	Dentures/dental manipulation
**Patient 5**	Female	46	22	No		No	Yes	12	Dentures/dental manipulation
**Patient 6**	Female	60	27	Abemaciclib		No	No	27	
**Patient 7**	Female	66	12	Palbociclib	17	DL, HTA	No	12	
**Patient 8**	Female	60	23	No		No	Yes	24	Dentures/dental manipulation
**Patient 9**	Female	56	24	No		No	No	24	
**Patient 10**	Female	60	15	No		No	No	15	
**Patient 11**	Female	61	6	Ribociclib	20	No	No	6	
**Patient 12**	Female	57	19	Palbociclib	25	No	No	20	
**Patient 13**	Female	83	13	No		DL, HTA, DM	Yes	13	Dentures/dental manipulation
**Patient 14**	Female	55	25	Ribociclib	29	No	No	28	Poor dental hygiene
**Patient 15**	Female	58	1	Palbociclib	17	DL	Yes	1	
**Patient 16**	Female	82	1	Abemaciclib	16	No	Yes	18	
**Patient 17**	Female	55	38	Abemaciclib	28	No	No	18	Dentures/dental manipulation
**Patient 18**	Female	60	11	No		No	Yes	12	
**Patient 19**	Female	48	23	No		No	No	29	Advanced periodontal disease
**Patient 20**	Female	83	11	Palbociclib	10	HTA, DL	No	11	Poor dental hygiene
**Patient 21**	Female	54	27	Abemaciclib	12	HTA	No	26	

## Data Availability

The data that support the findings of this study are openly available in Figshare at https://doi.org/10.6084/m9.figshare.24474865.v1 (accessed on 5 November 2023).
